# Exercise-induced muscle damage: multi-parametric MRI quantitative assessment

**DOI:** 10.1186/s12891-021-04085-z

**Published:** 2021-03-02

**Authors:** Xiaohong Lyu, Yue Gao, Qiang Liu, Heng Zhao, Huadong Zhou, Shinong Pan

**Affiliations:** 1 Department of Radiology, Shengjing Hospital of China Medical University, 36 Sanhao Street, Heping District, Shenyang, 110004 China; 2 Department of Radiology, The First Affiliated Hospital of Jinzhou Medical University, Jinzhou, 121000 China

**Keywords:** Rats, Eccentric exercise, Skeletal muscle damage, DTI, T2 mapping, IVIM

## Abstract

**Background:**

To explore the value of magnetic resonance quantitative analysis using diffusion tensor imaging, T2 mapping, and intravoxel incoherent motion in the evaluation of eccentric exercise-induced muscle damage and to compare the effects of various eccentric exercise modes at different time points in rats.

**Methods:**

A total of 174 Sprague-Dawley male rats were randomly divided into five groups: control, once-only exercise, continuous exercise, intermittent exercise, and once-fatigue exercise groups. Each experimental group was divided into seven time-subgroups: 0.5 h, 24 h, 48 h, 72 h, 96 h, 120 h and 168 h after exercise. The quadriceps femoris muscles were then scanned using magnetic resonance imaging. The apparent diffusion coefficient and fractional anisotropy values of diffusion tensor imaging, T2 values of T2 mapping, D and D* values of intravoxel incoherent motion and optical density values of desmin were measured. Associations among different eccentric exercise programmes, magnetic resonance imaging findings, and histopathological results were evaluated. Dunnett’s test, two-way repeated measures analysis of variance, and Pearson correlation analysis were used for statistical analysis.

**Results:**

Diffusion tensor imaging showed that the number of muscle fibre bundles decreased to varying degrees with different time points and eccentric exercises. Apparent diffusion coefficient values of the exercise groups showed a trend that first increased and then decreased, the opposite of fractional anisotropy. The specimens in all eccentric exercise programmes showed high signal T2 values after exercise, the highest among which was in the once-fatigue exercise group. D and D* in the experimental groups were significantly higher than those in the control group at 0.5–48 h after exercise. The apparent diffusion coefficient, fractional anisotropy, T2, D and D* values correlated with the optical density values of desmin.

**Conclusions:**

Diffusion tensor imaging, T2 mapping, and intravoxel incoherent motion technology accurately reflect the degree of skeletal muscle damage and recovery associated with eccentric exercise. The degree of muscle damage was the lowest in the continuous exercise group and the highest in the once-fatigue exercise group, which may provide more information and guidance for the formulation of physical and athletic training programmes.

## Background

Exercise-induced muscle damage (EIMD) commonly occurs after intensive and unaccustomed centrifugal exercises, such as strenuous endurance and strength training, and is characterised by delayed-onset muscle soreness (DOMS), muscle tenderness, muscle stiffness, muscle weakening, and volume enlargement [1–3]. EIMD can seriously affect living, working, and performing fitness exercise in the general population, as well as in sports training and professional sports competitions [4, 5]. Currently, the primary mechanisms of EIMD include mechanical injury, metabolism disorder, inflammation, and connective tissue injury theory [6–9]; the international mainstream view is that EIMD is attributed to the lack of homogeneity in the stretching of the sarcomeres, which is the mechanical damage of physical stress upon the muscle fibre [7]. This mechanical damage subsequently triggers the loss of calcium homeostasis, the production of reactive oxygen species, pro-inflammatory responses, oxidative stress and immune responses [10]. If the muscle tension remains the same while the muscle length changes, then the muscle contraction is isotonic; the muscle length can increase to produce eccentric contractions or shorten to produce concentric contractions. Eccentric exercises are more effective than concentric exercises [11] and are consequently the main mode that results in EIMD [12], the model for which was created by Armstrong [13].

Desmin is a type of muscle-specific intermediate fibrin that is mainly distributed in the cytoplasm, which is the primary component of the skeletal muscle, myocardium, and smooth muscle cells of vertebrates. It is a crucial index for research on the cytoskeleton and repair of the skeletal muscle damage, as well as an important structure to maintain the integrity of muscle cells. The loss of desmin after eccentric exercises has been proven to be a prelude to skeletal muscle damage [14, 15]. Although histological techniques can be used to determine muscle damage, the invasiveness of biopsies limits their applicability.

Owing to its better soft-tissue resolution and multi parameter imaging characteristics, magnetic resonance imaging (MRI) provides important information for evaluating the extent and degree of muscle damage. However, the conventional imaging examination lacks sensitivity to early muscle lesions. Diffusion tensor imaging (DTI) is a technology to quantitatively evaluate diffusion anisotropy of water molecules from multiple directions, which can provide information on the organisational proliferation and direction of the fibre bundles [16, 17]. Currently, DTI has been widely used in the field of study of the skeletal muscle and the nervous system [18, 19]. T2 mapping can reflect specificity through the detection of transverse magnetisation attenuation of tissues. T2 values are obtained by measuring the MR signal intensity of different echo times. T2 mapping is primarily applied to the bone and heart; more research has been performed on the joints and cartilage [20, 21], and less research has been conducted on muscles. Intravoxel incoherent movement (IVIM) is an imaging technique [22] that measures changes in blood perfusion [23]. However, limited reports exist on the application of IVIM for the diagnosis of muscle damage. Few studies have been conducted on exercise patterns and MRI evaluation methods for EIMD, and no studies have reported on MRI and clinical assessments under different eccentric exercise modes.

We hypothesised that different eccentric exercise modes would result in varying degrees of muscle damage. Hence, four eccentric exercise models based on human habitual motion patterns were applied in Sprague-Dawley rats, which is the most used animal model for studying EIMD. The aim of this study was to compare the effects of different time points, different eccentric modes, and relevance among variables using MRI quantitative parameters and histopathological expressions.

## Methods

### Group design

**Fig. 1:**
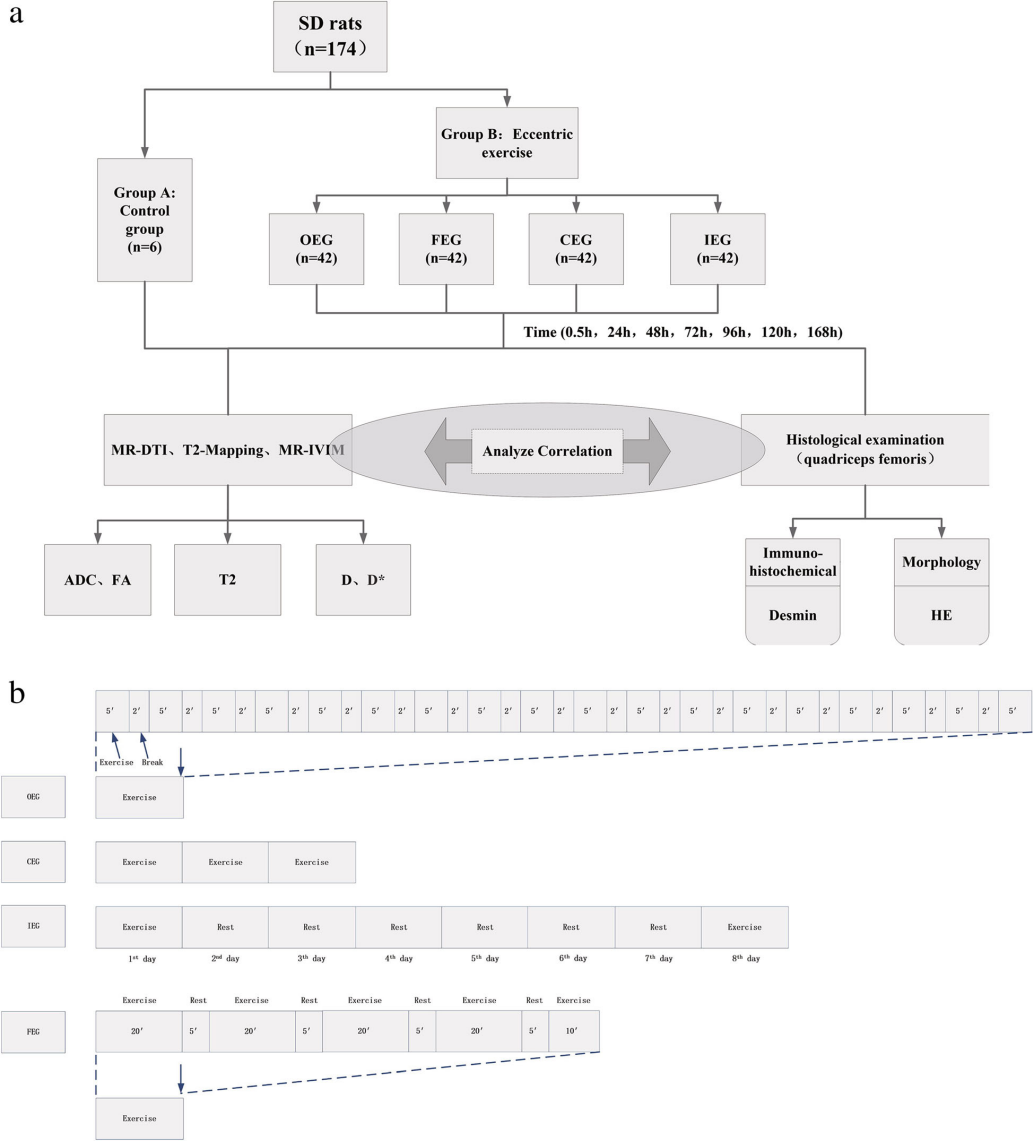
Flow chart (**a**) and exercise programmes (**b**) of the research

The experimental procedures and animal research were approved by the Ethics Committee of Shengjing Hospital of China Medical University (2019PS022K). A total of 174 male Sprague-Dawley rats, weighing 200 ± 20 g (specific-pathogen-free grade, aged 8 ± 0.6 weeks) were purchased from Changsheng Biotechnology Co., Ltd. (Liaoning, China). The sample sizes were determined using power analysis and the principles of 3Rs for animal research [24]. All rats were allocated individual cages with standard food and water in suitable environmental conditions (Animal Department, Benxi R & D Center, Shengjing Hospital, China). The rats were randomly divided into five groups: 1) control group (CTL, *n* = 6); 2) once-only exercise group (OEG, *n* = 42) which exercised once in 24 h; 3) continuous exercise group (CEG, *n* = 42) which exercised once in 24 h for 3 days (three bouts in total); 4) intermittent exercise group (IEG, *n* = 42) which exercised once, and then once more after a 6-day break (two bouts in total); and 5) once-fatigue exercise group (FEG, *n* = 42) which exercised only once with a different exercise protocol. Each experimental group was divided into seven subgroups according to the time points of the last exercise [10, 25]: 0.5 h, 24 h, 48 h, 72 h, 96 h, 120 h, and 168 h (Fig. 1a).

### Exercise programmes

Rats in CTL did not exercise, whereas those in the experimental groups were exercised to become familiar with treadmill running for 3 days at 09:00 am (10 min/day, 8–10 m/min). Three groups (OEG, CEG, and IEG) underwent the following eccentric exercise program: downhill treadmill running at a slope of − 16, a 5-min adaptation exercise at a speed of 8 m/min, and 5 min of running at a speed of 16 m/min with a 2-min rest, repeated 18 times. FEG performed treadmill running at a speed of 16 m/min for 20 min with a 5-min rest, repeated four times, and then running for another 10 min. Rats in all experimental groups exercised for a total of 90 min (Fig. 1b). Rats were stimulated with a weak electrical or photic charge to initiate running.

### MRI scanning

A 3.0 T superconducting MRI (Ingenia, Philips Healthcare, Best, Netherlands) and an eight-channel coil array (Chen Guang Medical Science and Technology Co, Ltd., Shanghai, China) were used for scanning. The rats were anaesthetised by intraperitoneal injection of pentobarbital sodium (30 mg/kg, Benxi R & D Center) after modelling and then placed in the prone position in the coil, with the femurs located in the centre of the coil. Conventional T1WIs, T2WIs, DTI, T2 mapping, and IVIM were obtained for the entire femur. The DTI parameters were as follows: TR/TE = 2500/63; slice thickness = 2.5 mm; slice spacing = 0.5 mm; 15 directions; b values (0, 600 s/mm^2^); and NSA = 2. The T2 mapping parameters were as follows: TR = 1000 ms; FOV = 13; matrix = 224 × 224; slice thickness = 2 mm; slice spacing = 0.2 mm; NEX = 0.5. The IVIM parameters were as follows: TR/TE = 2700/70; slice thickness = 2.5 mm; slice spacing = 0.5 mm; FOV = 100 × 80; VOXEL = 1.56 × 1.83 × 2.50; and 12 b values (0, 10, 20, 40, 60, 80, 100, 400, 600, 800, 1000, 1500 s/mm^2^). After MRI scanning, the rats were sacrificed by excessive intraperitoneal injection of pentobarbital sodium (200 mg/kg, Benxi R & D Center) and placed in the designated position of the experimental centre.

### MRI analysis

**Fig. 2:**
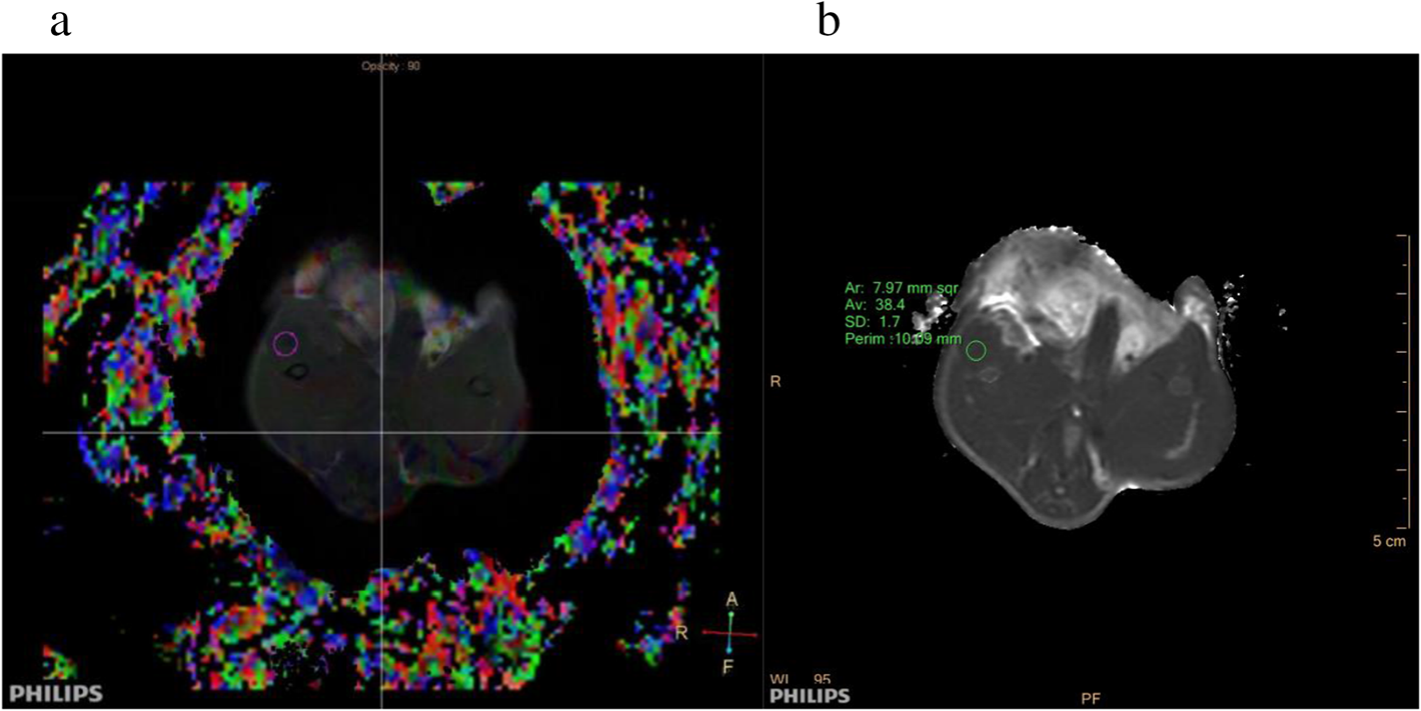
Regions of interest (ROI) of diffusion tensor imaging (**a**) and T2 mapping (**b**)

The apparent diffusion coefficient (ADC), fractional anisotropy (FA), and T2 values were measured using post-processing workstation (Ingenia, Philips, Netherlands); D and D* values were measured using IVIM post-processing software (v3.4, Philips, Netherlands), which were fitted to the mono-fitting method [26]. T1WI and DTI images were fused (Fig. 2a). The last sequence of T2 mapping was chosen for the measurements (Fig. 2b). Three-dimensional images of the quadriceps muscle were obtained via a morphological assessment: diffusion tensor tractography (DTT), and which is derived from DTI [27]. Two observers (S.N.P., with 29 years of experience in muscle MRI diagnosis; and Y.G., with 4 years of experience in muscle MRI diagnosis, including 5 months of MRI post-processing training) were trained to measure all the quantitative data twice, with a measurement interval of 4 weeks. They independently measured the whole data.

The measurement of the maximum region of quadriceps femoris injury was determined using T1WI axial anatomical and T2WI coronary positions. The regions of interest (ROI) were manually plotted around the lesions; the bones and blood vessels were excluded. The post-processing software automatically generated quantitative data and three-dimensional images of the muscle fibre within the scope of ROI. Measurements in the control group were conducted at the same location. Three measurements were conducted, and the results averaged.

### Histopathological examinations

After MRI, tissue specimens from the quadriceps muscles were taken, promptly put into 4% paraformaldehyde to avoid destruction and fixed at room temperature (20 ± 5 °C) for 1 week. The muscle specimens were then dehydrated, removed, and embedded in paraffin. The paraffin blocks were sectioned and dewaxed, and haematoxylin-eosin (HE) and desmin immunohistochemical staining were performed. HE staining was used to observe morphological changes and degree of damage to the skeletal muscle fibres. Image-Pro Plus 6.0 (Media Cybernetics, MD, USA) was used for optical density (OD) values of desmin.

### Statistical analysis

SPSS 19.0 software (IBM, Chicago, IL, USA) was used for the analysis. Homogeneity and consistency tests were conducted on the data. Descriptive data were expressed as mean ± standard deviation (SD). Inter- and intra-observer reliabilities for the measured data were calculated using the intraclass correlation coefficient (ICC). Dunnett’s test was performed to compare the ADC, FA, T2, D, D*, and OD values at each time point of the control versus experimental group. Two-way analysis of variance (ANOVA) was used to analyse differences of variables between the groups and different time points of the exercise mode, before which the Shapiro-Wilk test was performed for the data homogeneity test of variance. Finally, Pearson correlation analysis was performed, with OD values of desmin as the independent variable and ADC, FA, T2, D, and D* values as the dependent variables. Data were considered significant for *p*-value < 0.05.

## Results

### Inter- and intra-observer repeatability measures

The ICCs for the parameters (ADC, FA, T2, D, and D*) were higher than 0.75, indicating substantial or excellent measurement reliability (Table 1).

**Table 1:** Inter- and intra-observer reliability of quantitative parameters

Parameter	Inter-observer Reliability	Intra-observer Reliability	
		Observer 1	Observer 2
ADC	0.943	0.971	0.929
T2	0.905	0.921	0.893
FA	0.933	0.939	0.947
D	0.874	0.928	0.864
D*	0.881	0.824	0.906

ICC values ranged from 0 to 1, less than 0.4 indicating poor reliability, and greater than 0.75 indicating good reliability

*ADC* apparent diffusion coefficient, *T2* transverse relaxation time, *FA* fractional anisotropy, *D* true diffusion coefficient; D* pseudo-diffusion coefficient

### ADC, FA values of DTI and three-dimensional muscle fibre bundle of DTT in different groups

**Fig. 3:**
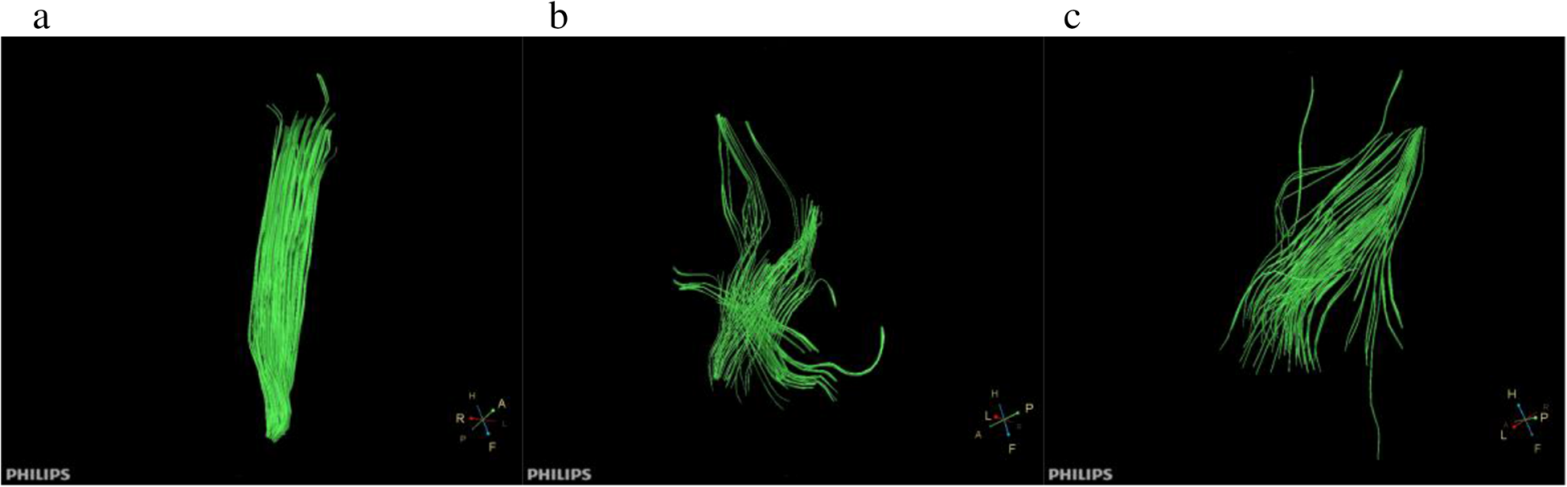
Diffusion tensor tractography of the quadriceps femoris. **a** represents control group (CTL); **b**, **c** represent 48 h, 120 h after exercise in the fatigue exercise group (FEG)

**Fig. 4:**
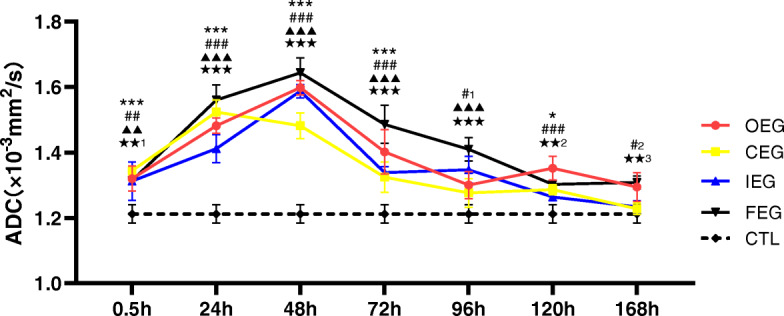
Changes in the apparent diffusion coefficient (ADC) values at each time point of different exercise modes and control group (CTL), analysed using Dunnett’ s test. *P = 0.020, ***P < 0.001, continuous exercise group (CEG) vs CTL; #P_1_ = 0.029, #P_2_ = 0.049, ##*P* = 0.004, ###*P* < 0.001, once-only exercise group (OEG) vs CTL; ▲▲*P* = 0.001, ▲▲▲*P* < 0.001, intermittent exercise group (IEG) vs CTL; ★★P_1_ = 0.001, ★★P_2_ = 0.002, ★★P_3_ = 0.009, ★★★*P* < 0.001, fatigue exercise group (FEG) vs CTL

**Fig. 5:**
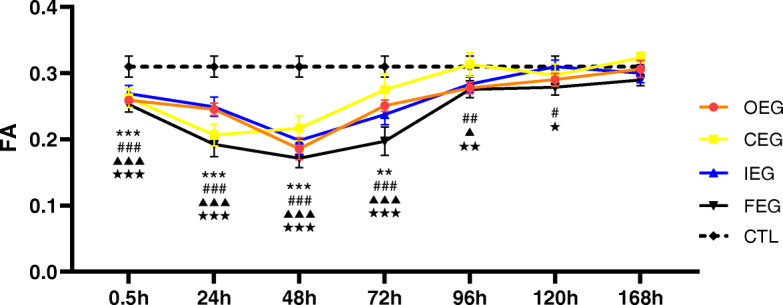
Changes in the fractional anisotropy (FA) values at each time point of different exercise modes and control group (CTL), analysed using Dunnett’s test. **P = 0.002, ***P < 0.001, continuous exercise group (CEG) vs CTL; #*P* = 0.029, ##*P* = 0.001, ###*P* < 0.001, once-only exercise group (OEG) vs CTL; ▲*P* = 0.026, ▲▲▲*P* < 0.001, intermittent exercise group (IEG) vs CTL; ★*P* = 0.014, ★★*P* = 0.005, ★★★*P* < 0.001, fatigue exercise group (FEG) vs CTL

Morphological assessment of DTT revealed that the injured muscle fibres were disorganised, distorted, and decreased in number compared with those in CTL (Fig. 3a, b, c). ADC values increased at 0.5 h after exercise, and the peak time and degree differed between exercise programmes. The values then declined to varying degrees (Fig. 4), and the trend of FA values was the opposite of that of ADC (Fig. 5). The highest ADC value and the lowest FA value appeared at 48 h of FEG. The amplitude and degree of variation in the ADC and FA values were the least in CEG, and the peak values appeared at 24 h.

### T2 values of T2 mapping in different groups

**Fig. 6:**
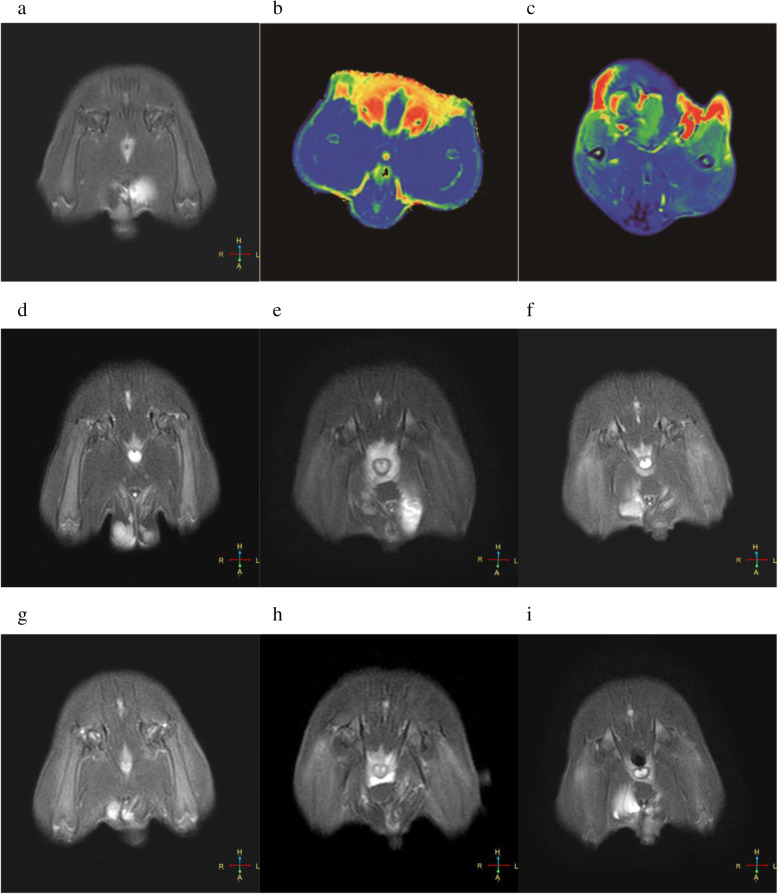
Coronal T2WI spectral adiabatic inversion recovery (SPAIR) of the femur showing changes in the signal of the quadriceps femoris at different time points after exercise in the fatigue exercise group (FEG) and continuous exercise group (CEG), and pseudo colour charts. **a**, **b** represent control group (CTL); **c** represents 48 h after exercise in the FEG; the green areas represent high signals; **d**, **e**, **f** represent 0.5 h, 24 h, and 48 h after exercise in the FEG; **g**, **h**, **i** represent 0.5 h, 24 h, 48 h after exercise in the CEG

**Fig. 7:**
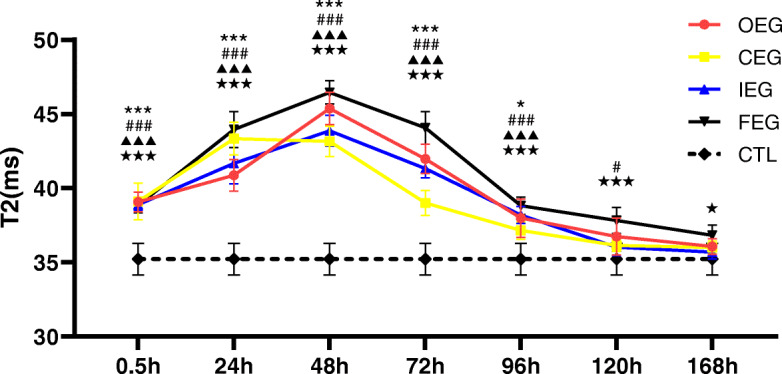
Changes in the T2 values at each time point of different exercise modes and control group (CTL), analysed using Dunnett’s test. *P = 0.032, ***P < 0.001, continuous exercise group (CEG) vs CTL; #*P* = 0.037, ###*P* < 0.001, once-only exercise group (OEG) vs CTL; ▲▲▲*P* < 0.001, intermittent exercise group (IEG) vs CTL; ★*P* = 0.048, ★★★*P* < 0.001, fatigue exercise group (FEG) vs CTL

Compared with CTL (Fig. 6a, b), the colour of the pseudo colour chart of quadriceps femoris changed (Fig. 6c); the signal intensity and T2 values increased at 0.5 h and 24 h in the FEG (Fig. 6d, e), OEG, and IEG peaking at 48 h (Fig. 6f) varying differing degrees (FEG > OEG > IEG, Fig. 7). At 120 h after exercise, the T2 values were close to normal in IEG, and after 168 h, the values approached normal in the OEG and those in the FEG were still slightly higher than in the controls. The trend was different in the CEG: the T2WI signal intensity and T2 values increased slightly after exercise, peaked at 24 h, and then declined (Figs. 6g, i, 7), to approach normal values at 96–120 h.

### D and D* values of IVIM in different groups

**Fig. 8:**
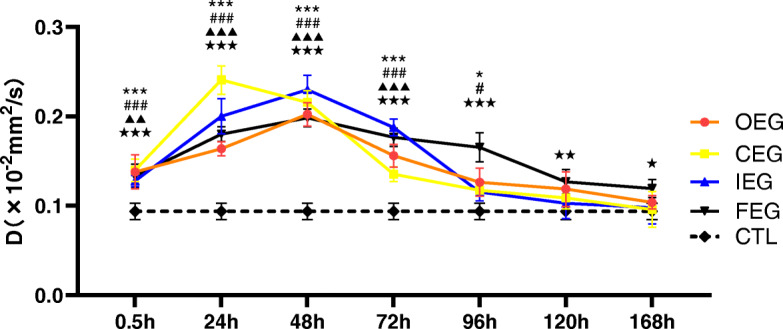
Changes in the D values at each time point of different exercise modes and control group (CTL), analysed using Dunnett’s test. *P = 0.022, ***P < 0.001, continuous exercise group (CEG) vs CTL; #*P* = 0.015, ###*P* < 0.001, once-only exercise group (OEG) vs CTL; ▲▲*P* = 0.006, ▲▲▲*P* < 0.001, intermittent exercise group (IEG) vs CTL; ★*P* = 0.028, ★★*P* = 0.002, ★★★*P* < 0.001, fatigue exercise group (FEG) vs CTL

**Fig. 9:**
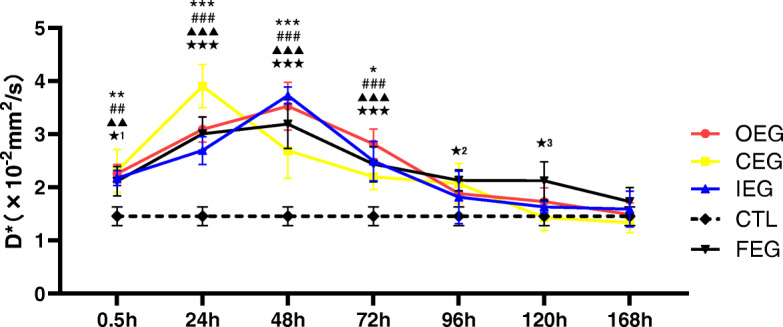
Changes in the D* values at each time point of different exercise modes and control group (CTL), analysed using Dunnett’s test. *P = 0.030, **P = 0.008, ***P < 0.001, continuous exercise group (CEG) vs CTL; ##*P* = 0.004, ###*P* < 0.001, once-only exercise group (OEG) vs CTL; ▲▲*P* = 0.008, ▲▲▲*P* < 0.001, intermittent exercise group (IEG) vs CTL; ★★P_1_ = 0.036, ★P_2_ = 0.029, ★P_3_ = 0.033, ★★★*P* < 0.001, fatigue exercise group (FEG) vs CTL

D and D* in the experimental groups first increased (0.5–48 h) and then declined. During 0.5–72 h after exercise, the D and D* values were significantly higher in the experimental groups than in CTL (Figs. 8 and 9). The D and D* values were the highest at 24 h of CEG. No significant difference was noted in D and D* values after 120 h between CTL and OEG, CEG, and IEG.

### Two-way repeated measures ANOVA analysis in different groups and time points

The interaction between group and time had a significant effect on ADC, FA, T2, D, D*, and desmin OD values. The results of the single effect test revealed statistically significant main effects of four exercise modes and time effects on each variable. Table 2 shows the resultant values of each variable.

**Table 2:** Two-way repeated measures analysis of variance in different groups and time points

Source		df	Mean Square	F	Sig.
ADC	GROUP	3	0.214	81.212	.000
	Error (GROUP)	12	0.003		
	TIME	1.359	.451	70.342	.000
	Error (TIME)	5.436	.006		
	GROUP ×TIME	2.177	.119	12.873	.002
	Error (GROUP×TIME)	8.706	.009		
FA	GROUP	3	.023	34.312	.000
	Error (GROUP)	12	.000		
	TIME	1.724	.076	61.920	.000
	Error (TIME)	10.898	.000		
	GROUP ×TIME	3.028	.009	10.169	.001
	Error (GROUP×TIME)	12.111	.001		
T2	GROUP	3	24.803	39.814	.000
	Error (GROUP)	12	.623		
	TIME	3.100	22.751	62.754	.000
	Error (TIME)	5.161	0.843		
	GROUP ×TIME	2.844	33.134	5.279	.017
	Error (GROUP×TIME)	11.377	6.276		
D	GROUP	3	.014	46.586	.000
	Error (GROUP)	12	.000		
	TIME	2.781	.056	78.101	.000
	Error (TIME)	11.112	.000		
	GROUP ×TIME	3.161	.012	13.711	.000
	Error (GROUP×TIME)	12.645	.001		
D*	GROUP	3	.132	5.031	.003
	Error (GROUP)	12	.044		
	TIME	1.783	31.079	34.734	.000
	Error (TIME)	7.134	.895		
	GROUP ×TIME	2.261	4.335	9.679	.005
	Error (GROUP×TIME)	9.043	.448		
Desmin	GROUP	3	.002	26.056	.000
	Error (GROUP)	12	0.001		
	TIME	2.418	.011	66.607	.000
	Error (TIME)	9.671	0.001		
	GROUP ×TIME	2.883	.002	5.151	.018
	Error (GROUP×TIME)	11.533	.000		

*ADC* apparent diffusion coefficient, *FA* fractional anisotropy, *T2* transverse relaxation time, *D* true diffusion coefficient, D* pseudo-diffusion coefficient

### HE staining changes

**Fig. 10:**
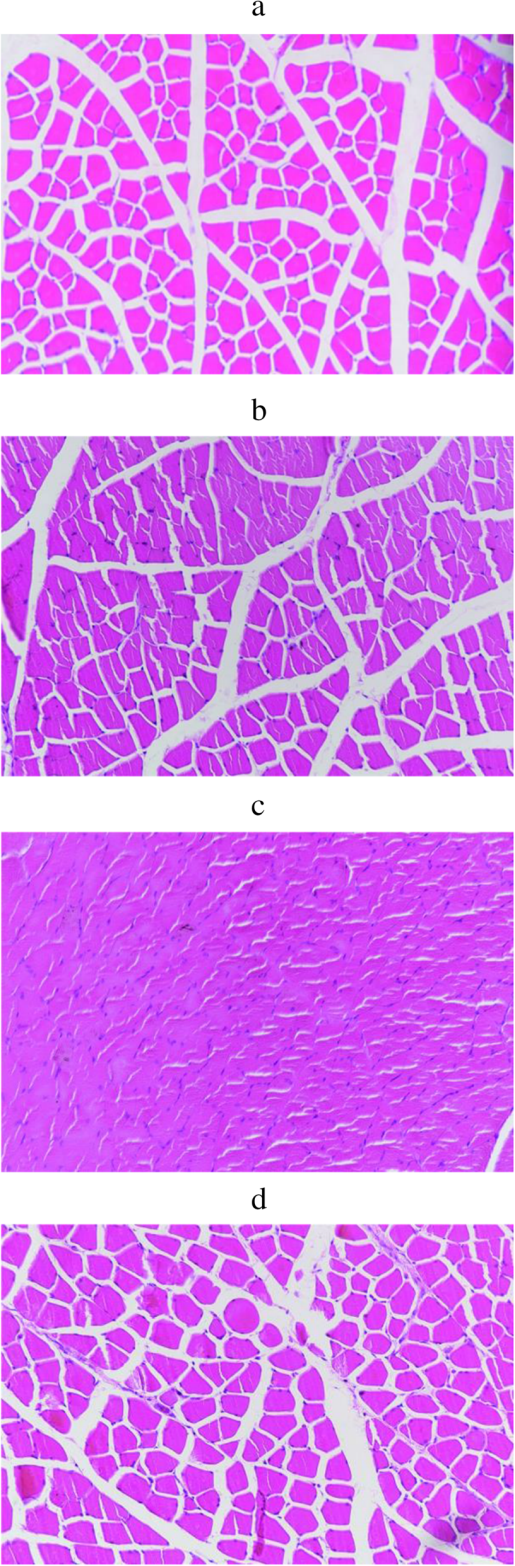
HE staining of the quadriceps femoris (X200). **a** represents control group (CTL); **b**, **c**, **d** represent 0.5 h, 48 h, 120 h after exercise in the fatigue exercise group (FEG)

HE staining showed that the muscular spaces were uniform without inflammatory infiltration and that the fibres were regularly arranged in CTL (Fig. 10a). The muscle fibres in the experimental groups were distorted, disorganized, and decreased in number; the extracellular spaces were enlarged, along with inflammatory infiltration. The changes were most obvious with part of the muscle fibres broken at 48 h after exercise in the FEG (Fig. 10b, c). At 120 h after exercise, the inflammatory infiltration decreased and the muscle fibres regenerated in the experimental groups (Fig. 10d). The recovery times were different in the CEG, IEG, OEG, and FEG—120 h, 120–144 h, 144 h, and more than 144 h, respectively.

### Immuno-histochemical results

**Fig. 11:**
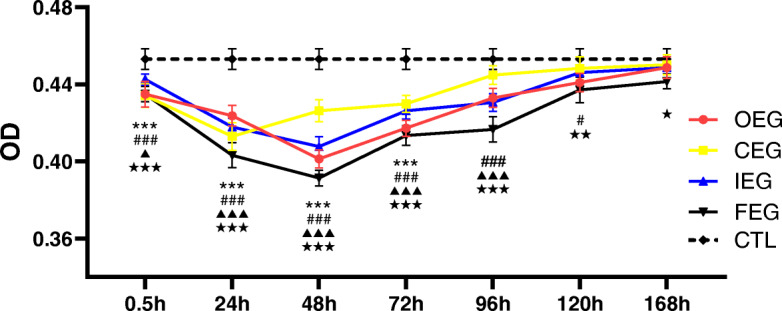
Changes in the optical density (OD) values of desmin at each time point of different exercise modes and control group (CTL), analysed using Dunnett’s test. ***P < 0.001, continuous exercise group (CEG) vs CTL; #*P* = 0.020, ###*P* < 0.001, once-only exercise group (OEG) vs CTL; ▲*P* = 0.019, ▲▲▲*P* < 0.001, intermittent exercise group (IEG) vs CTL; ★*P* = 0.033, ★★*P* = 0.001, ★★★*P* < 0.001, fatigue exercise group (FEG) vs CTL

Desmin staining of CTL was brown and distributed around the muscle fibres in groups, with an OD value of 0.453 ± 0.012. The lowest OD value was 0.391 ± 0.011, which appeared in the FEG at 48 h. The staining was light yellow, scattered, and absent around the muscle fibres. When comparing the lowest OD values in each experimental group, the highest value appeared in the CEG (0.413 ± 0.016); desmin staining was yellowish brown, sparsely distributed, and partially absent around the muscle fibres. The OD values increased by varying degrees during 96–168 h in all the experimental groups (Fig. 11).

### Correlation coefficients of ADC, FA, T2, D, D* values, and desmin expressions

We analysed the correlation coefficients to determine whether the ADC, FA, T2, D, and D* values correlated to desmin expressions. The correlation coefficients of ADC, FA, T2, D, D*, and OD values were − 0.871, 0.828, − 0.860, − 0.774, and − 0.752, respectively (Table 3), indicating that they correlated with desmin expression (*P* < 0.001).

**Table 3:** Pearson correlation coefficients between desmin and ADC, FA, T2, D, D* values

Parameters	ADC	FA	T2	D	D*
r	−0.871	0.828	−0.860	−0.774	−0.752
95% CI	(−0.906, −0.826)	(0.763,0.877)	(−0.900, −0.817)	(−0.822, −0.721)	(−0.815, −0.684)
P	<0.001	<0.001	<0.001	<0.001	<0.001

*ADC* apparent diffusion coefficient, *FA* fractional anisotropy, *T2* transverse relaxation time, *D* true diffusion coefficient, D* pseudo-diffusion coefficient

## Discussion

The present study demonstrated that the quantitative parameters of DTI, T2 mapping, and IVIM were consistent with changes in HE staining and desmin expressions in the muscle tissues. The baseline values of our ADC, FA, and D* in our study was 1.22 ± 0.03 × 10^− 3^ mm^2^/s, 0.31 ± 0.02, and 13.16 ± 1.26 × 10^− 3^ mm^2^/s, respectively, which are consistent with the results of previous studies [28, 29]. Baseline D value was 0.99 ± 0.11 × 10^− 3^ mm^2/^s, which is slightly lower than that reported in Jungmann’s study [29]. Baseline T2 value (35.22 ± 1.15 ms), as well as the maximum values in the OEG, were higher than those reported in Fu’s study [30]. We considered that the differences are due to the different technical parameters of magnetic resonance and modelling. The diffusion encoding parameters (b values and diffusion gradient directions), signal-to-noise ratio, and T2 changes can impact the quantitative indices [31]. In addition, the difference of animal species, fibre type [32], injury modelling (exercise programme), feeding pattern, and adaptive training affects the degree of muscle injury, thereby affecting the maximum values of the quantitative indices. EIMD can trigger a temporary increase in microcirculation and water content within the muscle [33, 34], as well as a decrease or disruption of the muscle fibres. DTI and fibre tracing technology can define the structure of muscle fibres in detail. Sinha et al. [35] found that DTI can show the direction and measure the length and cross-sectional area of muscle fibres. The structure of normal skeletal muscle fibre is orderly and complete. The influence of the cell membrane, transmembrane concentration gradient, and free diffusion in a direction perpendicular to the long axis of the muscle fibres makes the diffusion motion of water molecules slower than that of the parallel direction. Hence, the skeletal muscle is anisotropic. When the skeletal muscle is damaged, the diffusion of water molecules inside and outside the skeletal muscle cells changes; the oedema of the interstitium affects changes in DTI parameters such as ADC and FA values, reflecting anisotropy [18, 36]. Our study found that the ADC values increased at 0.5 h after eccentric exercises, peaked at 24–48 h, and then declined; the trend of FA values was the opposite of that of ADC. During repeated eccentric exercises, the diameter of the muscle fibre increased and the extracellular matrix and muscle fibre bundle were remodelled. Furthermore, destruction of the muscle fibres led to reduction of the diffusion restriction of water molecules, which increased the extracellular stroma space and the ADC value [37]. Yoon et al. [38] suggested that the FA value of skeletal muscle was related to fibre diameter, structural integrity, and density. The FA value increases when the arrangement order of the anisotropic tissue structure is regular and parallel, whereas it decreases when anisotropy of the fibres increases [39], which is consistent with our results. Fibre tracing showed that fibre shape changed in many directions after skeletal muscle injury, which can allow visual observation of changes in muscle fibre bundles. Clearly, DTI technology can objectively and accurately reflect the damage and recovery of skeletal muscle after exercise.

T2 mapping relies on increasing MRI transverse relaxation time [21, 40]. After muscle exercise, due to the increase of water molecules in and out of muscle cells, the relaxation time of protons and T2 values change [41]. Our study found that all eccentric exercises showed a delayed increase in the T2 values, which may relate to the delayed increase of enzymes in the muscle or increased concentration of serum myosin heavy chain fragments. The increase in T2 values is closely related to serum fsTnI level [30], cell lysis, necrosis, inflammatory reaction and myocyte regeneration in muscle; the peak T2 value is usually the period when there is more serious oedema and inflammatory reaction. Esposito et al. [42] demonstrated a statistical correlation between the injury score used to evaluate the degree of inflammatory response in the muscle damage and T2 values. Our study confirmed that the T2 values and trends are correlated with the expression of cytoskeletal protein desmin, the loss of which is a prelude to sarcomere changes triggered by eccentric exercises [15]. In addition, peaks of the T2 values, were the lowest in the CEG and the highest in the FEG, respectively, consistent with the changes in HE staining and DTI in the muscle tissue. This means that the quantification of T2 values within a certain range can help in objectively measuring the relaxation time of T2 muscle tissue, exclusive of the influence of subjective factors. To some extent, T2 mapping can reflect the expression of inflammatory response in muscles and indirectly indicate the severity of muscle damage.

IVIM is equivalent to diffusion weighted imaging of multiple b values. The parameters D and D* values represent the response relative to the real state of organization and diffusion of water molecules and blood capillary net of microcirculation perfusion-related situation [29, 32], respectively. The current study showed the D and D* values of the four experimental groups had a delayed increase after eccentric exercise, and the highest values were found in CEG. Morvan et al. [43] believed increase in the D and D* values after exercise were due to temperature. However, we consider the increase to be due to interstitial oedema and increased microcirculation perfusion. The peak times of perfusion parameters in the experimental groups were different, as it takes a different time for the muscles to adapt to local requirements [44]. Moreover, along with muscle contractions, the blood vessel will be compressed and will take longer to fill. The present study considered the reason for higher D and D* values in the CEG and IEG to be that repeated centrifugal exercises make the muscles adapt and promote microvascular circulation. In contrast, muscle injuries in the FEG were serious, which affected the recovery time of blood vessel filling; however, it showed less statistical difference in the D* values in the late stage of EIMD, which indicated that microcirculation perfusion was not obvious. Above all, IVIM can reflect the blood perfusion and diffusion function in EIMD.

The trends in skeletal muscle damage were similar but with varying degrees in the four groups, showing an immediate onset, reaching a peak at 24–48 h, and alleviating or recovering at 120–168 h. Comparing the four eccentric exercise models, the FEG had the highest peak damage degree with long duration and recovery time, whereas the CEG showed earlier damage peaking time and lowest damage degree. Overall damage in the IEG was slightly better than that in the OEG. However, the CEG having a lower degree of damage is in disagreement with some previous studies [5]. We believe this is related to differences in adaptive training, total exercise time, rest time, and animal modelling. The reason for damage changes in the IEG and CEG at each time point being less than that in the OEG is the “repeated bout effect” (RBE). This means that following a second bout of exercise, the muscle recovery is faster than in the initial bout [45]. Pincheira at al [44]. reported that the RBE causes adaptations in the non-contractile elements of muscles. Lynn et al. [46] suggested that repeated exercise leads to remodelling of the skeletal muscle sarcomeres. Along with injury repair, the muscles enhance the ability to resist the damage, and the regenerated muscle fibres strengthen the ability to resist the tension, which may even help to protect the spinal cord [47]. Consistent with Ziaaldini [48], H Zhao, a member of our team, reported increased the mRNA expressions of superoxide dismutase isoenzyme in the continuous eccentric programmes [25], which may associate with enhancement of muscle oxidative adaptation. Moreover, our study also indicates that high-intensity fatigue training results in more obvious injuries and an extension of recovery time. One important reason for the increasing incidence of sports-related injuries is the lack of standardised healthy exercise and training modes [49]. We considered that eccentric exercise can improve muscle function and neural adaptation to some extent [10, 50, 51]. Especially, RBE in the CEG and IEG demonstrated that properly repeating the eccentric session is a useful recovery strategy in EIMD. Our research technically verified that continuous exercise without fatigue can strengthen the muscles, promote health, and avoid damage, providing a more reasonable exercise mode as a potential means of muscle injury prevention.

The limitations of this study were as follows. First, as this was an animal experiment, clinical human studies should be conducted before extrapolating these results to application. Second, we did not study the threshold of the relevant parameters; whether the imaging markers can make a quantitative diagnosis of the grading for EIMD will be the core of further studies. Another limitation was that the relatively long imaging time of the functional MRI sequences may limit its clinical application.

## Conclusions

In conclusion, the findings confirm that quantitative MRI accurately reflects the histopathological abnormalities, as well as effectively responds to the degree of skeletal muscle damage induced by eccentric exercise. DTI, T2 mapping, and IVIM techniques can be applied to analyse the EIMD through water molecule diffusion, anisotropy, and muscle inflammation and microcirculation perfusion, respectively. The degree of damage was the least in the CEG, which may provide valuable information for the formulation of physical and athletic training programmes.

## Data Availability

The analyzed data sets generated during the current study are available from the corresponding author on reasonable request.

## Abbreviations

ADC: Apparent diffusion coefficient
ANOVA: Analysis of variance
CEG: Continuous exercise group
CTL: Control group
DOMS: Delayed-onset muscle soreness
DTI: Diffusion tensor imaging
DTT: Diffusion tensor tractography
EIMD: Exercise-induced muscle damage
FA: Fractional anisotropy
FEG: Fatigue exercise group
HE: Haematoxylin-eosin
ICC: Intraclass correlation coefficient
IEG: Intermittent exercise group
IVIM: Intravoxel incoherent motion
MRI: Magnetic resonance imaging
OD: Optical density
OEG: Only exercise group
RBE: Repeated bout effect
ROI: Regions of interest
SD: Standard deviation
SPAIR: Spectral adiabatic inversion recovery

## References

[1] Croisier JL, Camus G, Deby-Dupont G, Bertrand F, Lhermerout C, Crielaard JM (1996). Myocellular enzyme leakage, poly morphonuclear neutrophil activation and delayed onset muscle soreness induced by sokinetc eccentric exercise. Arch Physiol Biochem.

[2] Harris-Love MO, Seamon BA, Gonzales TI, Hernandez HJ, Pennington D, Hoover BM (2017). Eccentric exercise program design: A periodization model for rehabilitation applications. Front Physiol.

[3] Hedayatpoura N, Izanlooa Z, Falla D (2018). The effect of eccentric exercise and delayed onset muscle soreness on the homologous muscle of the contralateral limb. J Electromyogr Kinesiol.

[4] Baumert P, Lake MJ, Stewart CE, Drust B, Erskine RM (2016). Genetic variation exercise-induced muscle damage: implications for athletic performance, injury and ageing. Eur J Appl Physiol.

[5] Hedayatpour N, Falla D (2012). Non-uniform muscle adaptations to eccentric exercise and the implications for training and sport. J Electromyogr Kinesiol.

[6] Cheung K, Hume P, Maxwell L (2003). Delayed onset muscle soreness treatment strategies and performance factors. Sports Med.

[7] Morgan DL (1990). New insights into the behavior of muscle during active lengthening. Biophys J.

[8] Mohammadi H, Afzalpour ME, Levary SH (2018). Response of creatine kinase and lactate dehydrogenase enzymes to rest interval between sets and set-repetition configuration during bouts of eccentric exercise. Interv Med Appl Sci.

[9] Duranti G, Ceci R, Patrizio F, Sgrò P, Di Luigi L, Sabatini S (2018). Chronic consumption of quercetin reduces erythrocytes oxidative damage: evaluation at resting and after eccentric exercise in humans. Nutr Res.

[10] Hody S, Croisier JL, Bury T, Rogister B, Leprince P (2019). Eccentric muscle contractions: risks and benefits. Front Physiol.

[11] Franz A, Behringer M, Nosaka K, Buhren BA, Schrumpf H, Mayer C (2017). Mechanisms underpinning protection against eccentric exercise-induced muscle damage by ischemic preconditioning. Med Hypotheses.

[12] Lee K, Ochi E, Song H, Nakazato K (2015). Activation of AMP-activated protein kinase induce expression of FoxO1, FoxO3a, and myostatin after exercise-induced muscle damage. Biochem Biophys Res Commun.

[13] Armstrong RB, Warren GL, Warren JA (1991). Mechanisms of exercise-induced muscle fibre injury. Sport Med.

[14] Fridén J, Kjorell U, Thornell LE (1984). Delayed muscle soreness and cytoskeletal alterations: an immune cytological study in man. Int J Sports Med.

[15] Fridén J, Lieber RL (1998). Segmental muscle fiber lesions after repetitive eccentric contractions. Cell Tissue Res.

[16] Hooijmans MT, Damon BM, Froeling M, Versluis MJ, Burakiewicz J, Verschuuren JJ (2015). Evaluation of skeletal muscle DTI in patients with duchenne muscular dystrophy. NMR Biomed.

[17] Berry DB, Regner B, Galinsky V, Ward SR, Frank LR (2018). Relationships between tissue microstructure and the diffusion tensor in simulated skeletal muscle. Magn Reson Med.

[18] Hata J, Mizuno S, Haga Y, Shimoda M, Kanai Y, Chiba K (2018). Semiquantitative evaluation of muscle repair by diffusion tensor imaging in mice. JBMR plus.

[19] Pang H, Sun H, Fan G (2019). Correlations between the trigeminal nerve microstructural changes and the trigeminal-pontine angle features. Acta Neurochir.

[20] Eijgenraam SM, Bovendeert FAT, Verschueren J, van Tiel J, Bastiaansen-Jenniskens YM, Wesdorp MA (2019). T2 mapping of the meniscus is a biomarker for early osteoarthritis. Eur Radiol.

[21] Albano D, Chianca V, Cuocolo R, Bignone R, Ciccia F, Sconfienza LM (2018). T2-mapping of the sacroiliac joints at 1.5 tesla: a feasibility and reproducibility study. Skelet Radiol.

[22] Le Bihan D, Breton E, Lallemand D, Aubin ML, Vignaud J, Laval-Jeantet M (1988). Separation of diffusion and perfusion in intravoxel incoherent motion MR imaging. Radiology.

[23] Xu XQ, Choi YJ, Sung YS, Yoon RG, Jang SW, Park JE (2016). Intravoxel incoherent motion MR imaging in the head and neck: correlation with dynamic contrast-enhanced MR imaging and diffusion-weighted imaging. Korean J Radiol.

[24] Arck PC (2019). When 3 Rs meet a forth R: replacement, reduction and refinement of animals in research on reproduction. J Reprod Immunol.

[25] Zhao H, Liu J, Pan S, Sun Y, Li Q, Li F (2013). SOD mRNA and MDA expression in rectus femoris muscle of rats with different eccentric exercise programs and time points. PLoS One.

[26] Zhang G, Wang S, Wen D, Zhang J, Wei X, Ma W (2016). Comparison of non-Gaussian and Gaussian diffusion models of diffusion weighted imaging of rectal cancer at 3.0 T MRI. Sci Rep.

[27] Oh J, Jung JY, Ko YJ (2018). Can diffusion tensor imaging and tractography represent cross-sectional area of lumbar multifidus in patients with LUMBAR spine disease?. Muscle Nerve.

[28] McMillan AB, Shi D, Pratt SJP, Lovering RM (2011). Diffusion tensor MRI to assess damage in healthy and dystrophic skeletal muscle after lengthening contractions. J Biomed Biotechnol.

[29] Jungmann PM, Pfirrmann C, Federau C (2019). Characterization of lower limb muscle activation patterns during walking and running with Intravoxel incoherent motion (IVIM) MR perfusion imaging. Magn Reson Imaging.

[30] Fu C, Xia Y, Meng F, Li F, Liu Q, Zhao H (2020). MRI quantitative analysis of eccentric exercise-induced skeletal muscle injury in rats. Acad Radiol.

[31] Froeling M, Nederveen AJ, Nicolay K, Strijkers GJ (2013). DTI of human skeletal muscle: the effects of diffusion encoding parameters, signal-to-noise ratio and T2 on tensor indices and fiber tracts. NMR Biomed.

[32] Adelnia F, Shardell M, Bergeron CM, Fishbein KW, Spencer RG, Ferrucci L (2019). Diffusion-weighted MRI with intravoxel incoherent motion modeling for assessment of muscle perfusion in the thigh during post-exercise hyperemia in younger and older adults. NMR Biomed.

[33] Carlier PG (2011). Skeletal muscle perfusion and oxygenation assessed by dynamic NMR imaging and spectroscopy. Adv Exp Med Biol.

[34] Marro KI, Hyyti OM, Vincent MA, Kushmerick MJ (2005). Validation and advantages of FAWSETS perfusion measurements in skeletal muscle. NMR Biomed.

[35] Sinha S, Sinha U, Edgerton VR (2006). In vivo diffusion tensor imaging of the human calf muscle. J Magn Reson Imaging.

[36] Longwei X (2012). Clinical application of diffusion tensor magnetic resonance imaging in skeletal muscle. Muscles Ligaments Tendons J.

[37] Okamoto Y, Kemp GJ, Isobe T, Sato E, Hirano Y, Shoda J (2014). Changes in diffusion tensor imaging (DTI) eigenvalues of skeletal muscle due to hybrid exercise training. Magn Reson Imaging.

[38] Yoon MA, Hong SJ, Ku MC, Kang CH, Ahn KS, Kim BH (2018). Multiparametric MR imaging of age-related changes in healthy thigh muscles. Radiology.

[39] Okamoto Y, Kunimatsu A, Miki S, Shindo M, Niitsu M, Minami M (2008). Fractional anisotropy values of calf muscles in normative state after exercise: preliminary results. Magn Reson Med Sci.

[40] Snoj Z, Vidmar J, Gergar M, Plut D, Salapura V (2020). T2 distribution profiles are a good way to show cartilage regional variabilities and cartilage insufficiency. Skelet Radiol.

[41] Mathur S, Vohra RS, Germain SA, Forbes S, Bryant ND, Vandenborne K (2011). Changes in muscle T2 and tissue damage after downhill running in mix mice. Muscle Nerve.

[42] Esposito A, Campana L, Palmisano A, De Cobelli F, Canu T, Santarella F (2013). Magnetic resonance imaging at 7T reveals common events in age-related sarcopenia and in the homeostatic response to muscle sterile injury. PLoS One.

[43] Morvan D (1995). In vivo measurement of diffusion and pseudo-diffusion in skeletal muscle at rest and after exercise. Magn Reson Imaging.

[44] Pincheira PA, Hoffman BW, Cresswell AG, Carroll TJ, Brown NAT, Lichtwark GA (2018). The repeated bout effect can occur without mechanical and neuromuscular changes after a bout of eccentric exercise. Scand J Med Sci Sports.

[45] Nausheen S, Moiz JA, Raza S, Shareef MY, Anwer S, Alghadir AH (2017). Preconditioning by light-load eccentric exercise is equally effective as low-level laser therapy in attenuating exercise-induced muscle damage in collegiate men. J Pain Res.

[46] Lynn R, Talbot JA, Morgan DL (1998). Differences in rat skeletal muscles after incline and decline running. J Appl Physiol.

[47] Hosseinzadeh M, Andersen OK, Arendt-Nielsen L, Madeleine P (2013). Pain sensitivity is normalized after a repeated bout of eccentric exercise. Eur J Appl Physiol.

[48] Ziaaldini MM, Koltai E, Csende Z, Goto S, Boldogh I, Taylor AW (2015). Exercise training increases anabolic and attenuates catabolic and apoptotic processes in aged skeletal muscle of male rats. Exp Gerontol.

[49] Zwolski C, Quatman-Yates C, Paterno MV (2017). Resistance Training in Youth: Laying the Foundation for Injury Prevention and Physical Literacy. Sports Health.

[50] Douglas J, Pearson S, Ross A, McGuigan M (2017). Chronic adaptations to eccentric training: a systematic review. Sports Med.

[51] McNeill C, Beaven CM, McMaster DT, Gill N (2020). Survey of eccentric-based strength and conditioning practices in sport. J Strength Cond Res.

